# High Prevalence of Platelet Function Disorders in Women Referred for Surgical Management of Refractory Heavy Menstrual Bleeding

**DOI:** 10.1111/hae.70016

**Published:** 2025-03-03

**Authors:** Alison Delaney, Evangelos I. Kritsotakis, Kevin Horner, Steve Kitchen, Jennifer Sedcole, Ted Baxter, Rhona M. Maclean, Michael Makris, Clare V. Samuelson

**Affiliations:** ^1^ Sheffield Haemophilia and Thrombosis Centre Royal Hallamshire Hospital Sheffield UK; ^2^ Laboratory of Biostatistics School of Medicine University of Crete Heraklion Greece; ^3^ School of Health and Related Research University of Sheffield Sheffield UK; ^4^ Department of Obstetrics and Gynaecology Royal Hallamshire Hospital Sheffield UK; ^5^ Department of Cardiovascular Science University of Sheffield Sheffield UK

**Keywords:** ablation, heavy menstrual bleeding, light transmission aggregometry, menorrhagia, platelet function disorder, whole blood impedance aggregometry

## Abstract

**Introduction:**

Heavy menstrual bleeding (HMB) is a common presenting symptom in women with bleeding disorders, yet haemostatic testing is sometimes overlooked, even when refractory HMB requires surgical intervention.

**Aim:**

To determine the prevalence of bleeding disorders in women referred for surgical management of HMB and investigate screening approaches for bleeding disorders in this population.

**Methods:**

Women with refractory HMB referred for surgical management were enrolled prospectively and underwent a detailed haemostatic investigation. The International Society on Thrombosis and Haemostasis Bleeding Assessment Tool (ISTH‐BAT) and PFA‐100 assay were interrogated as screening tools for bleeding disorders. Multiplate whole blood impedance aggregometry (WBIA) was compared to the current gold‐standard lumiaggregometry testing for platelet dysfunction.

**Results:**

Fifty women underwent laboratory testing. Sixteen percent (95% confidence interval [CI] 7.2%–29.1%) were diagnosed with platelet function defects based on persistently abnormal lumiaggregometry results. No other clinically significant abnormalities were diagnosed. Women were more likely to be diagnosed with platelet dysfunction if they had failed a greater number of prior therapies, particularly prior endometrial ablation. The ISTH‐BAT lacked diagnostic accuracy, even at the calculated optimal cutoff value, and PFA‐100 assay lacked sensitivity. Multiplate WBIA was inferior to lumiaggregometry for the detection of platelet function disorders, with sensitivity of 62.5% (95% CI 24.5%–91.5%) and specificity of 87.5% (95% CI 73.2%–95.8%).

**Conclusion:**

Study findings support platelet function analysis by lumiaggregometry in women with refractory HMB requiring surgery. Accurate diagnosis would allow targeted haemostatic therapy and implementation of additional perioperative safety measures if surgery is still required.

## 1 Introduction

Heavy menstrual bleeding (HMB) is a common presenting symptom in women with bleeding disorders [1]. It impacts significantly on physical and psychological health, and in women with bleeding disorders causes particular morbidity [2].

Detection of haemostatic abnormalities in women with HMB facilitates consideration of alternative haemostatic measures, potentially abrogating the need for surgical treatments that reduce fertility and risk injury [2, 3]. In almost 70% of women with bleeding disorders, nonsurgical measures may control symptoms [4]. If surgery is needed, diagnosing bleeding disorders permits safer periprocedural care, including preemptive haemostatic therapies and avoidance of neuraxial anaesthesia [5]. Despite this, bleeding disorders are often overlooked by gynaecologists managing HMB [6, 7, 8].

Although many studies have investigated bleeding disorder prevalence amongst women with HMB [9, 10], this study aimed to establish prevalence within the subgroup requiring surgery for HMB. Bleeding disorders may be more common in this group, acknowledging bleeding disorder patients may more likely fail conservative therapies including the intrauterine system [11].

This study also investigated the utility of the International Society on Thrombosis and Haemostasis Bleeding Assessment Tool (ISTH‐BAT) [12], Platelet Function Analyser 100 (PFA‐100) and Multiplate whole blood impedance aggregometry (WBIA) in this population. The ISTH‐BAT is a validated screening tool for von Willebrand disease (vWD) [12, 13, 14] and may be discriminatory for severe platelet function defects (PFDs) [15, 16, 17, 18], yet its value for milder defects is unclear [3, 19–21]. Light transmission aggregometry (LTA) is the gold standard diagnostic test for PFDs and may be combined with nucleotide analysis via luminometry, ‘lumiaggregometry’ [22, 23]. However, lumiaggregometry is time‐consuming and requires expertise, highlighting a need for alternative screening/diagnostic tools [23, 24]. Despite prior discouraging results for PFA‐100 [25, 26, 27], it has not been examined in this population. WBIA has also been proposed as a faster, less labour‐intensive assay [23].

We present a prospective observational study, investigating the prevalence of bleeding disorders in women referred for surgical management of HMB. This is the first report of Multiplate WBIA in this subgroup.

## 2 Methods

This study took place at a UK hospital with tertiary gynaecology services and a Haemophilia Comprehensive Care Centre. All study procedures were in accordance with EU Good Clinical Practice guidelines [28], and the Declaration of Helsinki [29], following Regional Ethics Committee authorisation (REC reference 15/YH/0291).

Women were eligible if aged ≥18 and listed for endometrial ablation or hysterectomy for HMB. Exclusion criteria were: known/suspected pelvic malignancy, known bleeding disorder, anticoagulant/antiplatelet usage or inability to consent. Women taking nonsteroidal antiinflammatory agents were eligible if testing was arranged ≥10 days off treatment.

Eligible women were identified by gynaecology or research staff in outpatient clinics. Recruitment ran from 2016 to 2018. Due to slower than expected recruitment, a target of 50 women was set. All participants provided written informed consent.

### 2.1 Clinical and Laboratory Assessment

Research team members elicited a structured medical history and ISTH‐BAT [12]. ISTH‐BAT scores were confirmed by the leading study haematologist. Prior treatment for HMB was recorded. Where the same treatment was retrialled, this counted as one treatment, but different contraceptive preparations counted separately.

Women were issued a Pictorial Bleeding Assessment Chart at baseline [30], but due to the low return rate (16%) these results are not presented.

Laboratory investigations included full blood count, blood group, coagulation screen (PT, APTT, Clauss fibrinogen), testing for vWD (vWF:Ag, vWF:Act assay by Rcof, FVIII:c assay), PFA‐100 with EPI/COLL and ADP/COLL cartridges, LTA and ATP release by lumiaggregometry and Multiplate WBIA.

Methods and reagents included: PT–Dade Innovin, APTT–Dade Actin FS, Clauss Fibrinogen–Dade Thrombin, vWF:Ag–Siemens, vWF:Act–Innovance and FVIII:c Chromogenic assay–Biophen VIII (Hyphen Biomed) performed on Sysmex CS5100. PFA‐100: Dade Collagen/Epi and Dade Collagen/ADP test cartridges. All reagents and analysers–Sysmex Milton Keynes, UK.

LTA and ATP release were measured on CHRONO‐LOG Model 700 Whole Blood/Optical Lumi‐Aggregometer (Chrono‐log corporation).

Platelet‐rich plasma (PRP) was prepared by collecting 18 mL whole blood using a 21 gauge needle and 20 mL syringe, immediately transferring to a 20 mL universal container, then inverting gently with 2 mL trisodium citrate dihydrate 0.109 M. After hand delivery, samples were centrifuged (190 × *g* for 10 min, plasma removed, the remainder centrifuged at 2000 × *g* for 10 min) to obtain platelet‐poor plasma to set 100% maximum light transmission. The platelet count of PRP was measured and no adjustment made if 200–600 × 10^9^/L. PRP was rested for 30 min and testing completed within 4 h.

ATP release was normalised by dividing total release by absolute platelet number (platelet count multiplied by volume) to give results standardised to nanomoles of ATP released per 10^8^ platelets.

LTA was measured against the agonists (final concentrations) ADP (1, 3, 5, 10 µM), collagen (chrono‐Par) (1, 2, 4 µg/mL), epinephrine (3, 30 µM), ristocetin (American Biochemical and Pharmaceuticals Ltd) (0.5, 0.75, 1.0, 1.25, 1.5 mg/mL) and arachidonic acid (Bio Data corporation) (0.25, 0.5, 1.0, 1.5 mM). The lowest concentration of each agonist (except ristocetin) was used, moving to higher concentrations if a threshold response of 50% was not achieved or disaggregation observed. For ristocetin, starting concentration of 1.0 mg/mL was used and adjusted depending on response. ATP release was measured using luciferin d‐luciferase reagent (chrono‐lume) against thrombin (1 U/mL) and collagen (5 µg/mL). All reagents–chrono‐log corporation unless otherwise stated. Reference ranges for agonists were constructed from the analysis of 20 normal subjects.

WBIA was measured using the Multiplate analyser (Roche Diagnostics) according to manufacturer's instructions, but lower concentrations of each agonist were additionally used aiming to improve sensitivity. Agonists were: ADP (ADP Test, 3.28 and 1.22 µM), collagen (COL Test, 1.22 and 0.65 µg/mL), ristocetin (Risto Test, 0.77 and 0.48 mg/mL), arachidonic acid (ASPI Test, 0.48 and 0.25 mM) and TRAP (TRAP Test 8.1 and 4.0 µm). All reagents–Roche Diagnostics. Samples were collected into 2 × 3 mL tubes containing >15 µg/mL recombinant hirudin (Hirudin Blood Tube for Multiplate analysis, Ref: 6675751001, Roche Products).

Test results (excepting investigational WBIA) were reviewed and available to clinicians. If initial results (including vWF levels) were normal then no repeat analysis was undertaken. Women with laboratory abnormalities were assessed by a haemostasis specialist to direct management. Where lumiaggregometry was repeated due to initial abnormal results (to establish a PFD diagnosis), WBIA Multiplate analysis was also repeated, with normal controls included for each.

Perioperative and follow‐up documentation were reviewed ≥12 months following surgery, including haemostatic measures, surgical blood loss, and complications. Excess blood loss (EBL) was noted when documented by the surgeon or quantified as >100 mL. Following ablation, women were issued the ePAQ‐MPH (Menstrual, Pain and Hormonal) [31] questionnaire to establish ongoing HMB symptom burden.

### 2.2 Statistical Analysis

Numerical data were summarised as mean with standard deviation (SD) or median with range depending on distribution skewness. Categorical data were presented as frequency counts and percentages. ISTH‐BAT score distributions were compared between patients with PFD diagnosis (reproducible abnormality on lumiaggregometry) and without using Wilcoxon rank sum (Mann‐Witney) test. Strength and direction of associations between LTA‐confirmed PFD diagnosis and abnormal findings on PFA‐100, Multiplate and ISTH‐BAT were quantified using odds ratios (ORs) with 95% confidence intervals (CIs), adjusting for age, previously failed ablation and EBL. Logistic regression with penalised maximum likelihood estimation (Firth's method) was applied, accounting for a small sample size [32].

To evaluate the abilities of PFA‐100, Multiplate and ISTH‐BAT to discriminate between patients with or without PFD diagnosis, sensitivity, specificity, positive predictive value (PPV) and negative predictive value (NPV) were calculated at meaningful cutoffs. The optimal ISTH‐BAT cutoff value for PFD screening was taken as that which maximises Youden's index (sum of sensitivity and specificity). Data were analysed using STATA c.17 (StataCorp, TX, USA).

## 3 Results

Fifty‐four women with HMB referred for surgery were recruited. Fifty underwent ISTH‐BAT and laboratory investigations and are included in analyses (Table 1). Four were withdrawn by request or for nonattendance. Median age was 47 (range 26–56). Forty‐eight of the 50 had failed ≥1 treatment for HMB, with an average of three (range 0–7), including endometrial ablation in six (12%).

**TABLE 1:** Demographics and initial laboratory investigation results.

Variable	Summary statistics
Age (years), median (range)	47 (26.0–56.0)
ISTH BAT score, median (range)	6.0 (4.0–12.0)
Number of failed treatments (incl. ablation), median (range)	3.0 (0.0–7.0)
Tranexamic acid	33 (66.0%)
Mefenamic acid	16 (32.0%)
Oral progesterone	22 (44.0%)
Combined oral contraceptive pill	7 (14.0%)
Oral contraceptive unknown type	10 (20.0%)
Depo‐provera	10 (20.0%)
Intrauterine system	30 (60.0%)
GnRH analogue	5 (10.0%)
Ulipristal acetate	1 (2.0%)
Ablation	6 (12.0%)
Structural abnormality	27 (54.0%)
Adenomyosis alone	3 (6.0%)
Benign adenatoid tumour	1 (2.0%)
Endometrial polyp	1 (2.0%)
Endometrial stromal sarcoma–low grade	1 (2.0%)
Endometriosis	1 (2.0%)
Fibroids alone	15 (30%)
Fibroids and adenomyosis	3 (6.0%)
Fibroids and endometriosis	1 (2.0%)
Unicornuate endometrium	1 (2.0%)
Blood group	
A	24 (48.0%)
B	2 (4.0%)
O	20(40.0%)
Not reported	4 (8.0%)
Platelet count, median (range) (×10^9^/L)	279 (152–532)
Prothrombin time	
Normal	43 (86.0%)
Prolonged	7 (14.0%)
Activated partial thromboplastin time	
Normal	48 (96.0%)
Prolonged	2 (4.0%)
vWF:Ag, mean ± SD (range) (IU/mL)	1.2 ± 0.4 (0.5–2.1)
vWF:Rcof, mean ± SD (range) (IU/mL)	1.2 ± 0.4 (0.5–2.1)
FVIII:c, mean ± SD (range) (IU/mL)	1.5 ± 0.4 (0.7–2.8)
Lumiaggregometry initial	
Normal	37 (74.0%)
Abnormal	13 (26.0%)
Lumiaggregometry repeat following initial abnormal result	
Normal/decision not to repeat	5 (10.0%)
Abnormality confirmed	8 (16.0%)
Diagnosis of platelet function disorder made	
No	42 (84.0%)
Yes	8 (16.0%)

Abbreviations: FVIII:c, Factor VIII by coagulation assay; GnRH, gonadotropin releasing hormone; ISTH‐BAT, International Society on Thrombosis and Haemostasis Bleeding Assessment Tool; vWF:Ag, von Willebrand factor antigen; vWF:Rcof, von Willebrand factor activity.

### 3.1 Bleeding Disorder Prevalence

**FIGURE 1:**
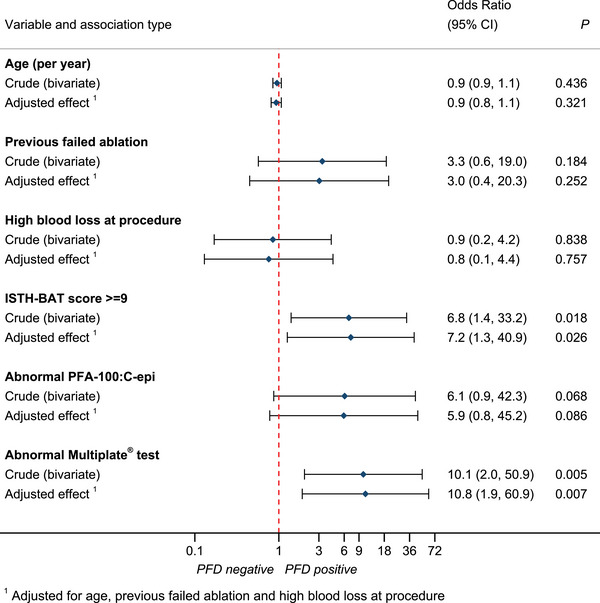
Forest plot of factors associated with diagnosis of platelet function disorder based on reproducibly abnormal results on lumiaggregometry testing. ISTH‐BAT, International Society on Thrombosis and Haemostasis Bleeding Assessment Tool.

Thirteen women (26%) had abnormal initial lumiaggregometry, and a PFD diagnosis was made in eight (16%, 95% CI 7.2%–29.1%) with repeatedly abnormal lumiaggregometry. Abnormalities included increased aggregation thresholds in two patients in response to ADP (1/8) or collagen (1/8), and reduced ATP release in six patients, with collagen alone (2/8), thrombin alone (2/8) or a combined defect to collagen and thrombin (2/8) (Tables S1 and S2). None had a reproducible aggregation and release defect. Three women with defective nucleotide release were diagnosed with storage pool disorder, whereas medication (selective serotonin reuptake inhibitors [SSRIs]) was possibly contributory in the other three (2/3 collagen alone, 1/3 thrombin alone). The median age of women diagnosed with a PFD was 46 (range 37–49). Age was not associated with PFD diagnosis or exclusion (Figure 1).

Women diagnosed with a PFD had undergone 1–5 prior HMB treatments. There was a suggestion of an increased likelihood of PFD with greater number of failed treatments, with OR 1.3 (95% CI 0.8–2.2) for each additional therapy. Women were more likely to be diagnosed with a PFD if they had HMB following ablation (OR 3.3, 95% CI 0.6–19.0) (Figure 1).

No other bleeding disorders were diagnosed. Platelet number, vWF:Ag, von Willebrand factor activity (vWF:Rcof), FVIII:c activity and Clauss fibrinogen were normal in all and not repeated. PT was prolonged in seven (14%) women, with two demonstrating mildly reduced FVII levels not considered clinically relevant. APTT was prolonged in two women. Factor IX was just below our reference range in one patient but not considered contributory to her symptoms; FXI was normal for both. Factor XII was normal in one of these patients but not tested in the other.

### 3.2 ISTH‐BAT and PFA‐100

**FIGURE 2:**
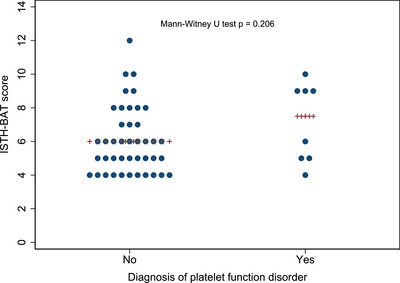
ISTH‐BAT score in women with and without a platelet function disorder based on repeatedly abnormal lumiaggregometry assay results. ++++ Represents the median score for each group. ISTH‐BAT, International Society on Thrombosis and Haemostasis Bleeding Assessment Tool.

The median ISTH‐BAT score was 7.5 (range 4–10) in women diagnosed with a PFD, compared to 6 (range 4–12) in women with normal platelet function. ISTH‐BAT cutoff value maximising sensitivity and specificity for PFD diagnosis (based on repeatedly abnormal lumiaggregometry) was calculated to be 8.5. ISTH‐BAT score of ≥9 was associated with a PFD diagnosis (OR 6.8, 95% CI 1.4–33.2, *p* = 0.018). However, there was a substantial overlap in score distributions between the two groups (Figure 2). If the cutoff of ≥9 was used, sensitivity was 50%, albeit estimated with much uncertainty (95% CI 15.7%–84.3%). By contrast, specificity was estimated to be 88.1% (95% CI 74.4%–96%). Dropping ISTH‐BAT cutoff to ≥7 reduced specificity to 69% without improving sensitivity. PPV of an ISTH‐BAT score ≥9 was 44.4% (95% CI 13.7%–78.8%) and NPV 90.2% (95% CI 76.9%–97.3%).

PFA‐100:C‐EPI was prolonged in four patients between 182 and 291s (normal range 79–161s). PFA‐100:C‐ADP was prolonged in one woman (217s, normal range 49–137s), who also had prolonged closure time with PFA‐100:C‐EPI. Of the four women with abnormal PFA‐100, only two (including the patient with prolongation in both cartridges) were diagnosed with a PFD. The other six women with a PFD had normal PFA‐100 closure times. Although abnormal PFA‐100:C‐EPI and PFA‐100:C‐ADP were specific for PFD (specificity 95.1% and 100%), sensitivity was low (25% and 12.5%).

### 3.3 Multiplate WBIA

Initial analysis with Multiplate WBIA was successfully completed in 48 (96%) women and was abnormal in 10 (20.8%). Abnormalities included low area under the curve (AUC) at high agonist concentration of collagen (3/48), arachidonic acid (2/48), TRAP alone (2/48), TRAP and ristocetin (2/48), and low AUC at low concentration ADP (1/48) and both ristocetin and ADP (1/48) (Table S1). One patient had abnormally low AUC for a high agonist concentration of TRAP and ristocetin and low AUC at a low agonist concentration of ADP and ristocetin–this patient had normal lumiaggregometry (Table S1).

Of the 10 patients with abnormal initial Multiplate results, five (50%) also had abnormal initial lumiaggregometry and had both repeated. Initial lumiaggregometry was normal in the other five (50%), therefore repeat investigation was not indicated. All five in whom abnormal initial Multiplate results were consistent with abnormal lumiaggregometry results went on to receive a diagnosis of PFD based on repeatedly abnormal lumiaggregometry. However, only 3/5 had abnormalities on repeat Multiplate investigation, with the other 2/5 having normal repeat Multiplate results (Table S2).

Initial Multiplate testing picked up 5/8 (62.5%) women with a PFD, and 3/8 women with a PFD (37.5%) had repeatedly abnormal Multiplate analysis. The sensitivity and specificity of initial Multiplate WBIA for PFD diagnosis in this population are 62.5% (95% CI 24.5%–91.5%) and 87.5% (95% CI 73.2%–95.8%), respectively. PPV of an initially abnormal Multiplate result for PFD diagnosis is 50% (95% CI 18.7%–81.3%) and NPV 92.1% (95% CI 78.6%–98.3%).

### 3.4 Structural Abnormalities

Twenty‐seven (54%) women had a structural pelvic abnormality (Table 1), with fibroids and/or adenomyosis being the most common (44%). Two of the 27 women (7.4%) with a structural abnormality were diagnosed with a concurrent PFD.

Patients with structural abnormalities had similar ISTH‐BAT scores (median [IQR], 5 [4–7] vs. 6 [5–9], *p* = 0.17) and number of failed treatments (median [IQR], 2 [2–3] vs. 3 [2–4], *p* = 0.15) compared to patients without. Structural abnormalities were not included in multivariate analyses as a confounding factor for PFD diagnosis, not being expected to impact lumiaggregometry results.

### 3.5 Surgical Outcomes

All eight women with a PFD proceeded to surgery, including Novasure endometrial ablation in 6/8 and laparoscopic hysterectomy in 2/8 (Table 2). Seven women with PFDs had additional preoperative haemostatic measures, including avoidance of neuraxial anaesthesia (7/7), tranexamic acid alone (4/7) and DDAVP with tranexamic acid (3/7). There were no perioperative complications in PFD patients.

**TABLE 2:** Surgical intervention and outcomes according to presence or absence of a platelet function disorder.

Surgery type	Platelet function disorder	Number	Excess blood loss	Postablation questionnaire results	Further treatment for menorrhagia required following ablation
			Number (%)	Number (%)	Number (%)
Endometrial ablation	Present	6	0 (0.0)	Improved: 4/6 (66.7)	2/6 (33.3)
				Not improved: 1/6 (16.7)	
				Not documented: 1/6 (16.7)	
	Absent	16	0 (0.0)	Improved: 7/16 (43.7)	6/16 (37.5)
				Not improved: 3/16 (18.8)	
				Not documented: 6/16 (37.5)	
Laparoscopic hysterectomy	Present	2	2 (100.0)	—	—
	Absent	14	6/14 (42.9)	—	—
Vaginal hysterectomy	Present	0	—	—	—
	Absent	1	1 (100.0)	—	—
Open hysterectomy	Present	0	—	—	—
	Absent	5	4/5 (80.0	—	—
Failed/unsuccessful treatment	Present	0	—	—	—
	Absent	3	0 (0.0)	—	—
Declined/EUA only	Present	0	—	—	—
	Absent	3	—	—	—

Abbreviation: EUA, examination under anaesthetic.

Women without PFDs underwent endometrial ablation (16/42), laparoscopic hysterectomy (14/42), vaginal hysterectomy (1/42) or open hysterectomy (5/42)–converted from laparoscopic in two.

There were no episodes of excessive surgical bleeding in women undergoing endometrial ablation. EBL was reported in 2/2 (100%) women with PFD undergoing laparoscopic hysterectomy, and 6/14 (42.9%) without PFD undergoing this procedure. There was no association between PFD diagnosis and EBL (OR 0.9, 95% CI 0.2–4.2, *p* = 0.838). EBL was more frequent in patients with structural abnormalities than those without (11/27 [41%] vs. 3/20 [15%]; OR = 3.9 [95% CI 0.9–16.6]; *p* = 0.056).

Of the six women with PFDs who underwent endometrial ablation, four reported improvement in HMB, whereas two required further treatment: progesterone‐only pill and tranexamic acid in one, hysterectomy in one.

For women without a PFD, improved bleeding postablation was reported by 7/10 (70%) for whom results were available; however, the ePAQ‐MPH questionnaire was not returned by 6/16 (37.5%). Six women without a PFD (37.5%) required additional treatment following ablation, a similar proportion to those with a PFD (33%).

## 4 Discussion

This study demonstrated a high prevalence of PFDs in women with HMB requiring surgical intervention. ISTH‐BAT, PFA‐100 and Multiplate WBIA lacked value as screening tools for PFDs, with low sensitivity (albeit large CIs); however, the small number of PFD cases detected means this study is underpowered to draw firm conclusions.

Sixteen percent of women in this study were diagnosed with platelet dysfunction. This is similar or lower than women with HMB reported to have bleeding disorders by other groups [3, 33–35], who found up to 47% to have PFDs [3, 35]. Surprisingly in this study which focussed on the select group requiring surgery, the proportion was not higher than the general HMB population. This may be because of the high average age of women in this study. Gynaecologists may be reluctant to recommend surgery in younger patients to preserve fertility. More severe bleeding disorders may have been diagnosed earlier and excluded, explaining why there were no diagnoses of severe PFDs or factor deficiencies.

However, it was expected that a number of vWD cases would be identified, the commonest bleeding disorder [16, 33, 34]. The lack of vWD diagnoses is likely related to a small sample size; however, since vWF levels increase with age, this diagnosis is less likely to be made in older women [36]. It is the authors’ opinion that vWD testing should be performed in all patients with HMB that is severe, associated with other bleeding symptoms (e.g., abnormal ISTH‐BAT score) or refractory to at least one conservative intervention.

Although women were diagnosed with a PFD based on lumiaggregometry, it is a limitation that further characterisation was not undertaken, for example with electron microscopy or genetic analysis. This did not impact on clinical management, but there are broader gains from accurate diagnoses, including improved patient understanding [35], access to support and family tracing.

Three women labelled as having a PFD were concurrently taking SSRIs, a recognised cause of platelet function abnormalities. It was impossible to quantify how these contributed to release defects without further investigation. Although not changing patient management, this limited the study's ability to establish inherited PFD prevalence.

Women diagnosed with a PFD had generally failed more conservative treatments and were more likely to have undergone prior ablation. A PFD was diagnosed in 7% with a structural pelvic abnormality, confirming the importance of haemostatic testing in all women with HMB requiring surgery [37].

Although an ISTH‐BAT score of ≥9 was associated with PFD diagnosis, within the limitations of small sample size and broad CIs, estimated sensitivity and PPVs at this threshold remained low. This concords with many published reports finding poor utility of ISTH‐BAT screening for milder disorders [3, 38]. It is clear from the range of ISTH‐BAT scores in patients without a PFD that those with normal haemostatic investigations still report bleeding symptoms (Figure 2).

PFA‐100 was proven to have no value as a screening tool for PFDs in this population, in agreement with numerous reports discouraging use due to poor sensitivity for milder disorders [25–27, 39].

Multiplate WBIA showed promise in early reports as an expeditious alternative to LTA for PFD diagnosis [39]. However, this study adds to growing evidence that it cannot be considered equivalent and cannot rule out mild PFDs in elective preoperative patients [40]. Had Multiplate been relied upon to exclude PFD in this cohort the diagnosis would have been missed in 62.5%.

No excess bleeding occurred following ablation in PFD patients; however, additional haemostatic measures were undertaken, potentially preventing this. No inferiority of outcome was demonstrated following ablation in women with PFDs despite a greater proportion having previously failed ablation. These data support the efficacy of ablation for women with PFDs; however, this would have been strengthened by quantitation of HMB pre‐ and post‐treatment.

## 5 Conclusion

Detailed haemostatic investigation should be recommended for all women with HMB meeting criteria for surgical intervention. This should include full blood count, coagulation screen and Clauss fibrinogen, vWD antigen and activity assays and LTA with nucleotide release studies, since PFA‐100 and Multiplate WBIA have poor sensitivity in this context.

A comprehensive assessment will improve the rate of diagnosis in women with HMB, allowing targeted haemostatic therapy to improve symptoms and optimise perioperative safety.

## Author Contributions

**Clare V. Samuelson**: devised and led the study, contributed to recruitment and data collection and, together with **Alison Delaney**, analysed data and prepared the manuscript. **Evangelos I. Kritsotakis**: provided statistical analysis of the data and interpretation of results. **Kevin Horner and Steve Kitchen**: performed and supervised laboratory testing in the study. **Jennifer Sedcole and Ted Baxter**: provided and analysed gynaecological operative outcome data. **Rhona M. Maclean and Michael Makris**: assisted with overall study design, interpretation of laboratory data and haemostatic management. All authors reviewed and approved the manuscript before publication.

## Ethics Statement

All study procedures were in accordance with EU Good Clinical Practice guidelines [30], and the Declaration of Helsinki [31], following Regional Ethics Committee authorisation (REC reference 15/YH/0291).

## Consent

All participants provided written informed consent.

## Conflicts of Interest

Alison Delaney has received speaker fees and worked on an advisory board for Kite Gilead. Alison Delaney has received speaker fees from Abbvie and Roche (unrelated to this work) and support for conference attendance from Kite Gilead, LFB Pharmaceuticals, Novo Nordisk, Hartley Taylor and Sheffield Hospitals Charity. Steve Kitchen has received consultancy/speaker fees from Sobi, Roche and Werfen. Michael Makris has provided consultancy to Novo Nordisk, Takeda, Sanofi and Grifols. Rhona M. Maclean has received honoraria/speaker fees from Sobi, Roche‐Chugai, Octapharma, Novo Nordisk, Bayer, Astra Zeneca and Janssen. Clare V. Samuelson has received advisory board and consultancy fees from Vertex, Agios. The other authors declare no conflicts of interest.

## Supporting information



Supporting Information

## Data Availability

Data pertaining to the PFA‐100, lumiaggregometry and Multiplate results that support the findings of this study are available in the Supporting Information of this article. Additional laboratory results data and gynaecological outcomes data are available from the corresponding author upon reasonable request.
