# Supplementary material for: High Prevalence of Platelet Function Disorders in Women Referred for Surgical Management of Refractory Heavy Menstrual Bleeding
Source: Haemophilia. 2025 Mar 3;31(3):485–93. doi: 10.1111/hae.70016 (PMC12175113; doi:10.1111/hae.70016)
Supplement: Supplementary file 1 — Supporting Information [file HAE-31-485-s001.docx]

**Supplementary table 1**. Results of initial platelet function investigations in all patients

| **Patient** | **PFA (sec.)** | | **Initial LTA (Threshold)** | | | | | **Initial ATP Release**  **(nm x10^8 plts/L)** | | **Initial Multiplate AUC at 6 min.**  **(high concentration)** | | | | | **Initial Multiplate AUC at 6 min.**  **(low concentration)** | | | | |
| --- | --- | --- | --- | --- | --- | --- | --- | --- | --- | --- | --- | --- | --- | --- | --- | --- | --- | --- | --- |
|  | **CADP** | **CEPI** | **ADP** | **COLL.** | **RISTO.** | **A.A.** | **EPI.** | **THR.** | **COLL.** | **ADP** | **ASP** | **COLL** | **RISTO** | **TRAP** | **ADP** | **ASP** | **COLL** | **RISTO** | **TRAP** |
|  | **49-137** | **79-161** | **<3** | **<1** | **0.5-1.0** | **<1.0** | **<30** | **0.73-1.8** | **0.78-2.14** | **>41** | **>65** | **>35** | **>42** | **>74** | **>21** | **>35** | **>12** | **>11** | **>-2** |
| 1 | 76 | **188** | **>10** | <1 | 0.75-1.0 | 0.25-0.5 | 3-30 | **0.62** | 0.8 | 104 | 131 | **15** | 132 | 114 | Failed | Failed | Failed | Failed | Failed |
| 2 | 102 | 136 | <1 | <1 | 0.75-1.0 | 0.25-0.5 | <3 | **0.45** | **0.73** | 94 | 121 | **33** | 86 | 71 | 96 | 114 | 17 | 60 | 50 |
| 3 | 72 | 67 | <1 | <1 | 0.75-1.0 | 0.25-0.5 | <3 | 1.01 | 1.81 | 68 | 93 | 52 | **10** | **22** | **12** | 91 | 45 | **10** | 16 |
| 4 | 75 | 114 | <1 | <1 | 0.75-1.0 | 0.25-0.5 | <3 | 1.04 | 1.28 | Failed | Failed | Failed | Failed | Failed | Failed | Failed | Failed | Failed | Failed |
| 5 | 69 | 122 | 1-3 | <1 | 0.75-1.0 | 0.5-1.0 | <3 | 0.98 | 1.22 | 67 | 98 | 62 | 91 | 84 | 29 | 86 | 26 | 12 | 17 |
| 6 | Failed | Failed | 1-3 | **1-2** | 0.75-1.0 | 0.5-1.0 | 3-30 | **0.42** | **0.7** | 68 | 70 | 59 | **35** | **67** | 66 | 60 | 43 | 13 | 37 |
| 7 | 104 | 117 | 1-3 | <1 | 0.75-1.0 | <0.25 | <3 | **0.42** | 0.91 | 57 | 90 | **32** | 98 | **22** | 38 | 117 | 31 | 28 | 23 |
| 8 | 86 | 94 | <1 | <1 | 0.5-0.75 | <0.25 | <3 | 1.02 | 1.25 | 100 | 111 | 79 | 109 | 113 | 65 | 120 | 63 | 97 | 63 |
| 9 | 77 | 99 | <1 | **1-2** | 0.5-0.75 | 0.25-0.5 | <3 | 1.2 | 0.99 | 60 | 85 | 38 | 62 | 84 | 49 | 73 | 22 | 49 | 60 |
| 10 | 87 | 115 | <1 | <1 | 0.5-0.75 | <0.25 | <3 | 1.19 | 1.42 | 84 | 84 | 56 | 114 | 74 | 64 | 94 | 31 | 69 | 19 |
| 11 | 76 | 101 | <1 | **1-2** | 0.5-0.75 | 0.25-0.5 | <3 | 0.97 | 1.4 | 83 | 106 | 74 | 108 | 116 | 40 | 88 | 61 | 57 | 75 |
| 12 | 90 | 126 | <1 | <1 | 0.75-1.0 | 0.25-0.5 | <3 | 0.8 | 1.03 | 69 | 89 | 70 | 96 | 89 | 45 | 71 | 34 | 13 | 26 |
| 13 | 77 | 94 | <1 | <1 | 0.75-1.0 | <0.25 | <3 | 0.85 | 1.11 | 71 | 106 | 48 | 104 | 96 | 32 | 79 | 29 | 44 | 52 |
| 14 | 87 | 89 | <1 | <1 | 0.75-1.0 | <0.25 | <3 | 1.05 | 1.52 | 75 | 120 | 56 | 127 | 127 | 46 | 121 | 12 | 124 | 55 |
| 15 | 85 | 118 | <1 | <1 | 0.75-1.0 | 0.25-0.5 | <3 | 1.13 | 1.3 | 91 | 91 | 75 | 114 | 114 | 101 | 108 | 85 | 48 | 14 |
| 16 | 120 | 145 | <1 | <1 | 0.5-0.75 | <0.25 | <3 | 1.11 | 1.29 | 89 | 108 | 73 | 101 | 115 | 76 | 100 | 45 | 96 | 106 |
| 17 | 78 | 97 | <1 | <1 | 0.5-0.75 | <0.25 | <3 | 0.83 | 1.24 | 98 | 109 | 87 | 158 | 105 | 57 | 104 | 72 | 26 | 45 |
| 18 | Failed | Failed | <1 | <1 | 0.75-1.0 | <0.25 | <3 | 1.57 | 1.86 | 103 | 90 | 72 | 109 | 93 | 68 | 83 | 65 | 63 | 91 |
| 19 | 122 | 155 | <1 | <1 | 0.75-1.0 | <0.25 | <3 | 1 | 0.93 | 79 | 102 | 50 | 123 | 104 | 63 | 102 | 18 | 12 | 27 |
| 20 | 87 | 126 | <1 | <1 | **1.0-1.25** | 0.25-0.5 | <3 | 0.87 | 0.88 | 76 | 85 | 67 | 123 | 106 | 45 | 71 | 71 | 47 | 88 |
| 21 | 87 | **182** | <1 | <1 | 0.75-1.0 | 0.25-0.5 | <3 | 1.13 | 1.53 | 120 | 122 | 86 | 154 | 125 | 106 | 132 | 95 | 108 | 114 |
| 22 | 72 | 86 | <1 | <1 | 0.5-0.75 | 0.25-0.5 | <3 | 0.97 | 0.9 | 114 | 135 | 77 | 172 | 131 | 96 | 120 | 26 | 75 | 77 |
| 23 | 100 | 123 | <1 | <1 | 0.5-0.75 | <0.25 | <3 | 0.93 | 0.99 | 93 | 93 | 69 | 115 | 130 | 73 | 112 | 61 | 97 | 82 |
| 24 | 114 | **190** | <1 | <1 | 0.5-0.75 | <0.25 | <3 | 0.94 | 1.08 | 153 | 165 | 141 | 262 | 171 | 157 | 161 | 17 | 51 | 129 |
| 25 | 82 | 110 | <1 | <1 | 0.75-1.0 | 0.25-0.5 | <3 | 1.18 | 1.69 | 96 | 107 | 51 | 133 | 111 | 73 | 93 | 25 | 33 | 32 |
| 26 | 91 | 129 | 1-3 | <1 | 0.75-1.0 | 0.25-0.5 | <3 | **0.61** | **0.68** | 74 | 93 | 59 | 100 | 90 | 77 | 97 | 56 | 48 | 31 |
| 27 | 137 | 130 | <1 | <1 | 0.75-1.0 | <0.25 | <3 | 1.17 | 1.45 | 83 | 115 | 65 | 138 | 120 | 94 | 122 | 75 | 72 | 112 |
| 28 | 103 | 126 | <1 | <1 | 0.75-1.0 | 0.25-0.5 | <3 | 1 | 1.08 | 116 | 107 | 92 | 120 | 119 | 92 | 101 | 64 | 13 | 99 |
| 29 | 119 | 109 | <1 | <1 | 0.75-1.0 | <0.25 | <3 | 1 | 0.98 | 92 | 115 | 82 | 146 | 109 | 95 | 123 | 62 | 18 | 38 |
| 30 | 79 | 126 | 1-3 | <1 | 0.5-0.75 | 0.25-0.5 | <3 | 1.12 | 1.22 | 65 | 70 | **33** | 98 | 78 | 58 | 82 | 29 | 107 | 77 |
| 31 | 112 | 135 | <1 | <1 | 0.5-0.75 | 0.25-0.5 | <3 | 1.36 | 1.23 | 85 | **63** | 65 | 102 | 109 | 61 | 64 | 12 | 56 | 27 |
| 32 | 85 | 95 | <1 | <1 | 0.5-0.75 | <0.25 | <3 | 1.28 | 1.38 | 109 | 112 | 89 | 121 | 120 | 101 | 104 | 86 | 113 | 114 |
| 33 | 97 | 136 | <1 | <1 | 0.75-1.0 | <0.25 | <3 | 1.06 | 1.15 | 103 | 116 | 84 | 135 | 126 | 89 | 113 | 59 | 47 | 114 |
| 34 | 78 | 108 | <1 | <1 | 0.75-1.0 | <0.25 | <3 | 1.07 | 1.09 | 110 | 100 | 55 | 148 | 106 | 101 | 117 | 23 | 28 | 109 |
| 35 | **217** | **291** | <1 | <1 | 0.75-1.0 | 0.25-0.5 | <3 | **0.58** | **0.76** | 110 | 120 | 72 | 172 | 121 | 65 | 115 | 78 | 135 | 53 |
| 36 | 103 | 86 | <1 | <1 | 0.5-0.75 | <0.25 | <3 | 1.19 | 1.29 | 115 | 133 | 92 | 172 | 133 | 85 | 126 | 103 | 144 | 104 |
| 37 | 92 | 120 | 1-3 | <1 | 0.5-0.75 | <0.25 | <3 | 0.93 | 0.78 | 76 | 126 | 92 | 172 | 179 | 56 | 131 | 79 | 94 | 94 |
| 38 | 121 | 131 | <1 | <1 | 0.5-0.75 | <0.25 | <3 | 1.19 | 1.42 | 104 | 114 | 82 | 127 | 121 | 99 | 120 | 77 | 98 | 111 |
| 39 | 78 | 97 | 1-3 | <1 | 0.75-1.0 | 0.25-0.5 | <3 | 1.34 | 1.69 | 103 | 131 | 102 |  | 148 | 76 | 106 | 72 | 90 | 103 |
| 40 | 118 | 114 | <1 | <1 | 0.75-1.0 | 0.25-0.5 | <3 | **0.51** | 0.94 | 91 | 111 | 76 | 113 | 100 | 76 | 93 | 47 | 72 | 52 |
| 41 | 106 | 115 | <1 | <1 | 0.5-075 | <0.25 | <3 | 1.04 | 1.16 | 96 | 124 | 94 | 152 | 130 | 95 | 129 | 104 | 113 | 52 |
| 42 | 93 | 156 | 1-3 | **1-2** | 0.75-1.0 | 0.25-0.5 | <3 | 1.07 | 1.07 | 98 | 747 | 90 | 126 | 170 | 86 | 147 | 85 | 68 | 121 |
| 43 | 89 | 95 | <1 | <1 | 0.75-1.0 | 0.5-1.0 | <3 | 0.75 | 0.88 | 90 | 77 | 70 | 81 | 101 | 85 | 64 | 62 | 21 | 90 |
| 44 | 87 | 105 | <1 | <1 | 0.75-1.0 | <0.25 | <3 | 0.81 | 1.26 | 82 | 86 | 64 | 74 | 105 | 77 | 90 | 64 | 82 | 90 |
| 45 | 125 | 152 | 1-3 | <1 | 0.75-1.0 | 0.5-1.0 | **>30** | 0.93 | **0.65** | 76 | **64** | 51 | 130 | 131 | 77 | 88 | 19 | 12 | 114 |
| 46 | 98 | 140 | 1-3. | <1 | 0.75-1.0 | 0.25-0.5 | <3 | 0.87 | 1.43 | 71 | 122 | 68 | 135 | **32** | 132 | 117 | 22 | 20 | 12 |
| 47 | 112 | 136 | <1 | <1 | 0.5-0.75 | <0.25 | <3 | 1.26 | 1.51 | 109 | 139 | 100 | 200 | 139 | 102 | 119 | 69 | 114 | 113 |
| 48 | 120 | 144 | <1 | <1 | 0.75-1.0 | 0.25-0.5 | <3 | 0.91 | 1.44 | 62 | 75 | 37 | 68 | 82 | 62 | 75 | 22 | 27 | 29 |
| 49 | 133 | 126 | 1-3. | **1-2.** | 0.75-1.0 | 0.5-1.0 | <3 | 0.75 | 1.42 | 363 | 396 | Failed | 509 | 411 | 76 | 77 | 154 | 37 | 53 |
| 50 | 88 | 110 | <1 | <1 | 0.5-0.75 | <0.25 | <3 | 1.16 | 1.56 | 72 | 105 | 139 | 105 | 107 | **11** | 108 | 149 | 103 | 101 |

*Agonists used and normal ranges in the top row.*

*Abnormal results highlighted in bold. Grey shaded rows represent those with initially abnormal lumiaggregometry that had repeat platelet investigations.*

*A.A., Arachidonic Acid; ADP, Adenosine diphosphate; ASP, ASPI Test (Roche) - Arachidonic Acid; ATP, Adenosine triphosphate; AUC, Area under the curve; CADP, Collagen and Adenosine diphosphate test cartridge; CEPI, Collagen and Epinephrine test cartridge; COLL, Collagen; EPI, Epinephrine; PFA, Platelet Function Analyser; RIST, Ristocetin; THR, Thrombin Receptor Activating Peptide.*

**Supplementary table 2**. Results of repeat platelet function investigations in those with abnormal initial lumiaggregometry

| **Patient** | **Repeat LTA (Threshold)** | | | | | **Repeat ATP Release**  **(nm x10^8 plts/L)** | | **Repeat Multiplate AUC at 6 min.**  **(high concentration)** | | | | | **Repeat Multiplate AUC at 6 min.**  **(low concentration)** | | | | |
| --- | --- | --- | --- | --- | --- | --- | --- | --- | --- | --- | --- | --- | --- | --- | --- | --- | --- |
|  | **ADP** | **COLL.** | **RISTO.** | **A.A.** | **EPI.** | **THR.** | **COLL.** | **ADP** | **ASP** | **COLL** | **RISTO** | **TRAP** | **ADP** | **ASP** | **COLL** | **RISTO** | **TRAP** |
|  | **<3** | **<1** | **0.5-1.0** | **<1.0** | **<30** | **0.73-1.8** | **0.78-2.14** | **>41** | **>65** | **>35** | **>42** | **>74** | **>21** | **>35** | **>12** | **>11** | **>-2** |
| 1 | **>10** | **1-2** | 0.75-1.0 | 0.5-1.0 | 3-30 | 0.86 | 1.21 | 102 | 117 | 38 | 131 | 124 | 40 | 76 | 15 | 26 | 24 |
| 2 | <1 | <1 | 0.75-1.0 | 0.25-0.5 | <3 | 0.74 | **0.54** | 112 | 114 | 60 | 121 | 124 | 68 | 118 | 18 | 25 | 17 |
| 6 | 1-3 | **1-2** | 0.75-1.0 | 0.25-0.5 | 3-30 | 0.81 | 0.91 | 86 | 92 | 56 | 82 | **58** | 55 | 71 | 27 | 36 | 21 |
| 7 | <1 | <1 | 0.75-1.0 | <0.25 | <3 | **0.49** | 0.99 | 48 | 68 | **28** | **12** | **16** | **19** | 58 | 65 | **5** | 10 |
| 9 | Clinical decision not to repeat | | | | |  |  |  |  |  |  |  |  |  |  |  |  |
| 11 | <1 | <1 | 0.75-1.0 | 0.25-0.5 | <3 | 1.85 | 1.43 | 69 | 116 | 79 | 42 | 111 | 44 | 99 | 30 | 26 | 35 |
| 20 | <1 | <1 | 0.75-1.0 | 0.5-1.0 | <3 | 1.01 | 1.14 | 91 | 115 | 82 | 118 | 128 | 88 | 101 | 75 | 44 | 89 |
| 26 | <1 | <1 | 0.75-1.0 | 0.25-0.5 | <3 | **0.57** | **0.73** | 80 | 96 | 49 | 75 | 78 | 58 | 93 | 34 | 66 | 16 |
| 35 | <1 | <1 | 0.5-0.75 | <0.25 | <3 | **0.69** | **0.65** | 73 | 91 | 53 | 144 | 95 | 65 | 85 | 61 | 60 | 70 |
| 40 | <1 | <1 | 0.75-1.0 | 0.25-0.5 | <3 | **0.7** | 0.94 | 68 | 102 | 60 | 100 | 87 | 48 | 79 | 43 | 74 | 20 |
| 42 | 1-3 | <1 | 0.75-1.0 | 0.25-0.5 | <3 | 1.37 | 1.19 | 97 | 128 | 75 | 145 | 135 | 59 | 115 | 65 | 40 | 84 |
| 45 | 1-3 | **1-2** | 0.75-1.0 | 0.5-1.0 | 3-30 | 0.81 | **0.56** | 93 | 117 | **23** | 191 | 142 | 78 | 52 | 23 | 182 | 127 |
| 49 | <1 | <1 | 0.75-1.0 | 0.5-1.0 | <3 | 0.74 | 1.22 | 106 | 116 | 97 | 145 | 80 | 50 | 93 | 54 | 29 | 34 |

*Agonists used and normal ranges in the top row.*

*Abnormal results highlighted in bold. Grey shaded rows represent patients diagnosed with a PFD in this study.*

*A.A., Arachidonic Acid; ADP, Adenosine diphosphate; ASP, ASPI Test (Roche) - Arachidonic Acid; ATP, Adenosine triphosphate; AUC, Area under the curve; CADP, Collagen and Adenosine diphosphate test cartridge; CEPI, Collagen and Epinephrine test cartridge; COLL, Collagen; EPI, Epinephrine; PFA, Platelet Function Analyser; RIST, Ristocetin; THR, Thrombin Receptor Activating Peptide*
